# Proteoglycans Determine the Dynamic Landscape of EMT and Cancer Cell Stemness

**DOI:** 10.3390/cancers14215328

**Published:** 2022-10-29

**Authors:** Zoi Karagiorgou, Panagiotis N. Fountas, Dimitra Manou, Erik Knutsen, Achilleas D. Theocharis

**Affiliations:** 1 Biochemistry, Biochemical Analysis & Matrix Pathobiology Research Group, Laboratory of Biochemistry, Department of Chemistry, University of Patras, 26504 Patras, Greece; 2 Department of Medical Biology, Faculty of Health Sciences, UiT the Arctic University of Norway, 9010 Tromsø, Norway; 3 Centre for Clinical Research and Education, University Hospital of North Norway, 9038 Tromsø, Norway

**Keywords:** proteoglycans, serglycin, versican, extracellular matrix, epithelial-to-mesenchymal transition, cancer stem cells

## Abstract

**Simple Summary:**

Proteoglycans are important structural and functional components of extracellular matrices that govern phenotype and functions of resident cells. They are often deregulated in cancer cells and their stroma, providing a favorable microenvironment for cancer cell growth and spread. Proteoglycans, by influencing cell–cell and cell–matrix interactions and oncogenic signaling, affect cancer cell phenotype and properties. In this article, we discussed the implication of proteoglycans with cancer cell stemness and epithelial-to-mesenchymal transition.

**Abstract:**

Proteoglycans (PGs) are pivotal components of extracellular matrices, involved in a variety of processes such as migration, invasion, morphogenesis, differentiation, drug resistance, and epithelial-to-mesenchymal transition (EMT). Cellular plasticity is a crucial intermediate phenotypic state acquired by cancer cells, which can modulate EMT and the generation of cancer stem cells (CSCs). PGs affect cell plasticity, stemness, and EMT, altering the cellular shape and functions. PGs control these functions, either by direct activation of signaling cascades, acting as co-receptors, or through regulation of the availability of biological compounds such as growth factors and cytokines. Differential expression of microRNAs is also associated with the expression of PGs and their interplay is implicated in the fine tuning of cancer cell phenotype and potential. This review summarizes the involvement of PGs in the regulation of EMT and stemness of cancer cells and highlights the molecular mechanisms.

## 1. Introduction

**Figure 1:**
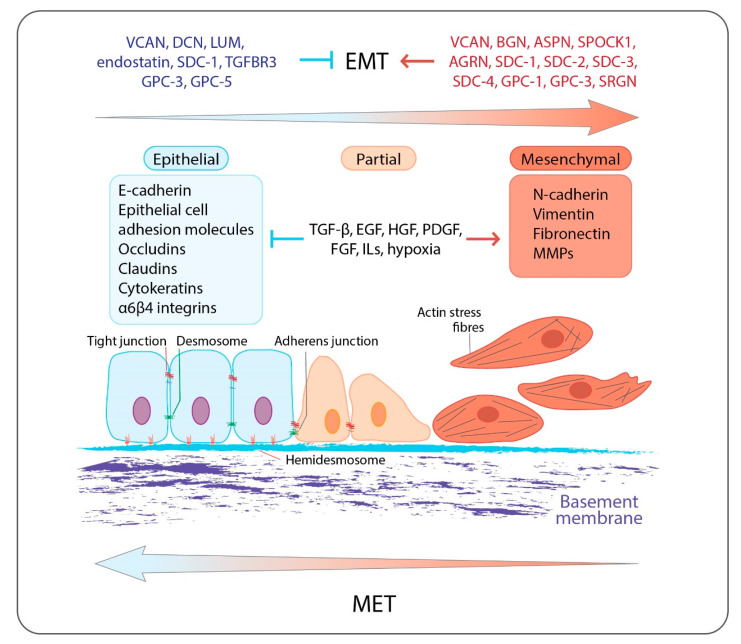
Overview of EMT: epithelial cells are anchored to the underlying basement membrane by hemidesmosomes and are interconnected by tight junctions, adherens junctions, and desmosomes. They express specific cell surface and cytoskeletal proteins associated with epithelial phenotype such as E-cadherin, epithelial cell adhesion molecules, occludins and claudins, epithelial cell-associated integrins α6β4, and cytokeratins. EMT is triggered by numerous growth factors, cytokines, and hypoxia, which activate a transcription program, which is associated with the inhibition of the expression of epithelial-related genes and the induction of the expression of mesenchymal-related genes including N-cadherin, vimentin, ECM molecules, fibronectin, and MMPs. Mesenchymal cells display increased motility and invasiveness. Epithelial cells during their progressive transition to a mesenchymal phenotype acquire an intermediate state (partial EMT), into which cells exhibit a mixed cell phenotype expressing both epithelial and mesenchymal markers. PGs are involved in the EMT program affecting, among others, EMT-related signaling pathways acting either as inducers or as inhibitors. EMT can be reversed where the mesenchymal cells can revert to the epithelial phenotype by undergoing MET. Both EMT and MET occur during cancer progression and the development of distant metastases.

Extracellular matrices (ECMs) are dynamic three-dimensional macromolecular networks which together with the embedded cells form complex structures called tissues, which are further assembled to create organs [1]. Besides acting as physical scaffolds, ECMs support all different cell tissue functions and constitute the main regulators of numerous cell properties, such as survival, proliferation, adhesion, migrations, differentiation, and apoptosis. The main macromolecules of ECMs are collagens, proteoglycans (PGs), laminins, fibronectin, elastin, other glycoproteins, and ECM-degrading enzymes such as proteinases [2]. PGs are crucial ECMs constituents, as they localize throughout the ECMs and are key mediators of cell behavior both in physiological and pathological conditions [3,4]. All PGs are composed of a core protein, which is decorated with variable number and type(s) of five different glycosaminoglycans (GAGs), named chondroitin sulfate (CS), dermatan sulfate (DS), keratan sulfate (KS), heparan sulfate (HS), and heparin (HP). As a result, a variety of PG editing can be achieved, with diverse interaction motifs and roles in the orchestration of cell signaling and phenotype [5,6]. Several members of the PG family have been shown to be deregulated during tumorigenesis and cell differentiation, leading to changes in epithelial-to-mesenchymal transition (EMT) (Figure 1) [5,7]. EMT is a cellular process during which epithelial cells repress their epithelial characteristics and acquire mesenchymal phenotypes and behavior [8,9]. The process has drawn much attention due to its association with cancer metastasis and resistance (Figure 1) [10,11,12].

EMT is an essential biological process under normal physiology, as it is involved in embryonic development and tissue regeneration and repair. In embryonic development, it takes part in gastrulation and renal, heart valve, and neural crest formation, while in wound healing EMT is mediated by inflammatory cells and fibroblasts, and provides the epithelial cells lining the wound with migratory properties so that they can repopulate the wound [13,14,15]. EMT is induced during normal and pathological conditions by a range of cytokines and growth factor-signaling pathways including transforming growth factor β (TGFβ), bone morphogenetic protein (BMP), interleukins (ILs), Wnt–β-catenin, Notch, Hedgehog, receptor tyrosine kinases, and hypoxia (Figure 1) [16]. The major reprogramming of the gene expression during EMT is conducted by a group of transcription factors (TFs) commonly referred to as EMT-TFs, including the Snail family (Snail, encoded by SNAI1, and Slug, encoded by SNAI2), the zinc finger E-box-binding homeobox (ZEB) family (ZEB1 and ZEB2), and the Twist family BHLH transcription factors (TWIST1 and TWIST2) [17,18], yet other transcription factors, such as paired-related homeobox 1 (PRRX1) and forkhead box C2 (FOXC2), can also induce EMT [19,20].

One of the main features of EMT is the acquisition of resistance to anoikis-apoptosis upon loss of attachment to the ECM and neighboring cells [21]. Anoikis comes from the Greek word meaning homelessness, and is a mechanism to prevent inappropriate translocation and attachment of cells in tissues. In the process of EMT, mesenchymal cells lose epithelial adherens junctions, rearrange their actin cytoskeleton, and alter their ECM interactions [8,9]. These changes allow the cells to become motile, and, in addition, the cells induce expression of proteins with properties that can modulate the ECM, such as matrix metalloproteinases (MMPs) (Figure 1) [8,22]. Epithelial cells, on the contrary, have a tight connection with the ECM. Integrins, cell adhesion molecules, and various adaptor proteins take part in these interactions, and bridge the ECM molecules with the cell cytoskeleton (Figure 1) [8,9]. The interconnections established between various ECM components as well as between matrix components with cells allow mechanical force and regulatory signals to be transmitted from the ECM to the cells [23]. For this reason, the ECM is of major importance for tissue homeostasis, and the composition, or changes in composition, of ECM molecules have a direct impact on the signaling networks of the cells. Cancer cells that have undergone EMT and other cells within the tumor stroma, such as tumor-associated macrophages (TAMs) and cancer-associated fibroblasts (CAFs), contribute to ECM degradation and remodeling to facilitate tumor cell plasticity [24]. Further, specific ECM–integrin interactions have been associated with changes in the EMT phenotypes of cells, an example being hyaluronan (HA), which has been shown to induce EMT via it its receptor, CD44 [25]. CD44 has been tightly linked to EMT, as it is upregulated by TGFβ, is directly involved with STAT3, β-catenin, and AKT signaling, and promotes tumor invasion and metastasis by contributing to the adhesion of cancer cells to the endothelium and fibronectin-enriched matrices [26]. CD44 is both considered to be a mesenchymal marker gene and also a marker gene for cancer stem cells.

In vivo experiments show that in breast cancer, but also other cancer types, only a subpopulation of the cancer cells have self-renewing capabilities and stem-like characteristics [27]. These cells have been termed cancer stem cells (CSCs), or also known as tumor-initiating cells. Several cell-surface markers have so far been proposed as makers of CSCs, including EMA, CALLA, CD49f, EpCAM, CD44, and CD24 [28], and many of these markers overlap with cells that have undergone EMT [27]. In addition, CSC express EMT transcription factors, suggesting that mechanisms regulating EMT and stemness are closely integrated [27]. It has been suggested that CSCs have a combined epithelial and mesenchymal phenotype, defined as epithelial mesenchymal plasticity (EMP), permitting these cells to move between an epithelial and a mesenchymal state [29]. EMT, EMP, and the acquisition of stem-like characteristics have all been linked to chemotherapy resistance, dormancy, and relapse [30,31,32]. ECM components, such as several collagens, glycoproteins, PGs, and HA are produced in high quantities by CSCs and take part in regulating their properties. Furthermore, the physical and mechanical properties of the ECM affect CSCs functions, and it has been suggested that a deeper understanding of the involvement of ECM components on cancer stemness could provide novel opportunities for therapies against CSCs [33].

## 2. Proteoglycans in Brief

PGs are classified into four categories by taking into consideration their localization, the extracellular, pericellular, cell surface, and intracellular states, and further subdivided based on their structural and functional characteristics [7,34,35].

The extracellular PGs are classified into three main subfamilies: the hyalectans, the small leucine-rich proteoglycans (SLRPs), and the Testican/SPARC/Osteonectin CWCV and Kazal-like domain (SPOCK).

Aggrecan, versican (VCAN), brevican, and neurocan are the four members that comprise the hyalectan family and are characterized by C-terminus and N-terminus globular domains that interact with a plethora of ECM molecules and a central part where GAGs, especially CS and KS, bind. Their distribution and functions are exceptionally diverse. For example, aggrecan is mainly found in cartilage and brain tissues, bridging different ECM molecules. The four isoforms of VCAN, V0, V1, V2, and V3 are located in the blood vessels, heart, breast, and brain tissues in diverse proportions, while brevican and neurocan are mainly expressed in the central nervous system. VCAN is a multifunctional molecule that affects cell adhesion, proliferation, and migration via binding to CD44, integrin-β1, and toll-like receptors (TLRs) as well as activating epidermal growth factor receptor (EGFR) signaling pathway by direct binding through its EGF-like domain present at the C-terminus globular domain [7,34,35].

SLRPs constitute a wide PGs family, with repeating leucine residues in their protein core, and are expressed in various tissues. SLRPs can be subdivided based on their structure and organization characteristics into five different classes: three canonical SLRP classes, types I–III, and two non-canonical classes, types IV and V. Decorin (DCN) and biglycan (BGN) belong to SLRP class I, and contain one and two CS/DS chains, respectively, whereas lumican (LUM) is a member of SLRP class II, and carries two to four KS chains. All three of them contain domains that interact with various growth factors (GFs) and GF receptors, such as vascular endothelial growth factor A (VEGFA), TGFβ, platelet-derived growth factor (PDGF), connective tissue growth factor (CTGF), EGFR, insulin-like growth factor receptor I (IGFR-I), VEGFR-II, as well as TLRs, modulating processes including cell growth, development, and inflammation [5,7,34,35].

The pericellular PGs family contains the following four members: Agrin (AGRN), perlecan/HSPG2, and collagen types XV and XVIII. All four members bear a combination of CS and HS chains attached to their diverse protein cores. CS and HS chains mediate the interactions of pericellular PGs with GFs as well as with inflammation and matrix remodeling-related molecules. Interestingly, the C-terminal domain of perlecan/HSPG2 and collagen type XVIII can be proteolytically cleaved, thereby generating bioactive fragments named endorepellin and endostatin, respectively [5,7,34,35].

The cell surface PG superfamily is mainly divided in two subclasses, syndecans and glypicans, whereas three more proteoglycans, CSPG4, phosphacan, and betaglycan, are enlisted in this superfamily. The family of syndecans contains four members of transmembrane PGs (syndecan 1–4), expressed in numerous cell types. Syndecans mainly carry HS chains [5]. They play crucial roles both in physiological processes, such as development, and in certain pathological conditions [5,7,36]. For example, altered expression levels of syndecans can lead to cancer progression, stemness, EMT, and metastasis. Glypicans consist of six members (glypican 1–6) of glycosylphosphatidylinositol (GPI)-anchored cell membrane PGs. They are mainly substituted with HS chains. Their main role is in the regulation of several signaling pathways such as Wnt, Hedgehog, fibroblast growth factor (FGF), TGFβ, and BMPs [5,7,37]. Lastly, betaglycan, also known as TGFβ receptor III (TGFBR3), is a co-receptor of the TGFβ family, regulating its ligands’ bioavailability. The role of TGFBR3 in EMT is highly relevant, as the TGFβ signaling pathway is a crucial modulator, both in physiological and pathological conditions [38].

Serglycin (SRGN) is the only PG that has been characterized as an intracellular PG. The core of SRGN contains a domain with repeating Ser–Gly residues, where eight CS, DS, HS, or HP chains can attach in a cell- and condition-dependent manner. SRGN is expressed by various cells such as hematopoietic, endothelial, smooth muscle cells, fibroblasts, and tumor cells. It can regulate the formation and maturation of secretory granules, and the secretion and the bioavailability of various growth factors, cytokines, and functional ECM molecules involved in tumorigenesis and inflammation. SRGN also directly stimulates oncogenic signaling in several tumor cell types [39,40].

**Figure 2:**
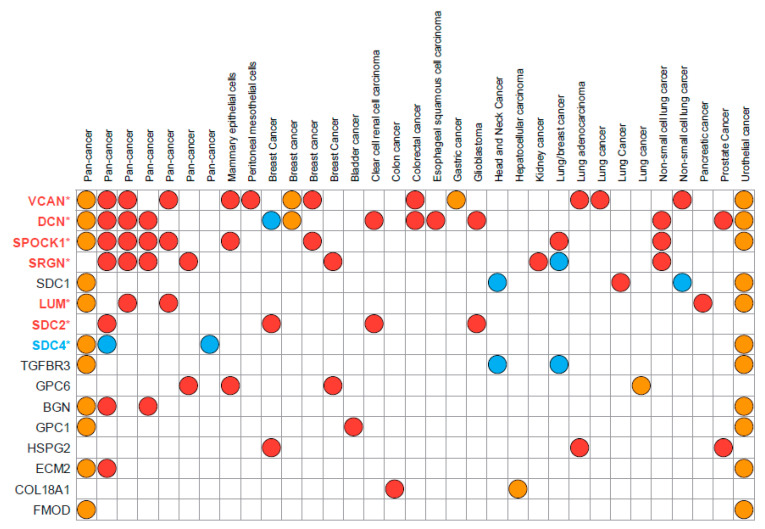
Proteoglycans found in the proposed EMT signatures deposited in the EMTome database. Eighty-four EMT signatures were downloaded from the EMTome database (http://www.emtome.org/ (accessed on 19 August 2022)) and searched for overlap with 42 proteoglycans. Only proteoglycans identified in at least two signatures are displayed in the figure. Red color indicates association towards the mesenchymal phenotype, blue towards the epithelial phenotype, and yellow indicates that no association towards either the mesenchymal or epithelial phenotype was found in the proposed dataset. Proteoglycans with stars after their name were also discovered as TGFβ-responsive genes in our own publication (PMID: 35494005) Here, colors of the name represent their association to either epithelial (blue) or mesenchymal (red) phenotypes.

PGs participate in cell–cell and cell–matrix interactions and oncogenic signaling affecting cancer cell phenotype and functional properties. Differential expression of PGs is associated with EMT in various cancer types, and multiple PGs have been identified in proposed EMT signatures deposited in the EMTome database (http://www.emtome.org/ (accessed on 19 August 2022)) [41] (Figure 2).

## 3. Proteoglycans as Regulators of EMT and Cell Stemness

### 3.1. Association of Versican with EMT

VCAN is an extracellular EMT mediator that affects cell phenotypes and functions. A crucial step for embryogenesis is the differentiation of pluripotent embryonic stem cells (ESCs) via embryoid bodies (EBs), a phenomenon driven by induction of EMT in ESCs, in which the expression and specific distribution of VCAN and HA are of great importance. VCAN is highly expressed by CSCs [33,42,43]. Suppressed VCAN expression blocks breast cancer cell self-renewal, while overexpression of the G3-domain of VCAN promotes cancer cell self-renewal via the EGFR/AKT/GSK3β pathway [42] (Table 1). In CD133+/CD44+ prostate CSCs, VCAN causes the suppression of cancer cell attachment to fibronectin and thereby increases cell motility [43]. VCAN is positively regulated by the EMT transcription factor Snail. In addition to VCAN, Snail induces transcription of the sulfate donor PAPSS2, and thus leads to elevated sulfation levels of VCAN, which in turn is associated with increased migration and metastasis [44]. Moreover, the role of VCAN in EMT during cancer seems to be isoform specific. For instance, in breast cancer cells, VCAN V2 expression suppressed Snail and induced E-cadherin protein levels through inhibition of the EGFR/ERK/GSK3β axis. On the contrary, VCAN V1 is proposed to enhance the EGFR pathway [45] (Table 1).

VCAN is also reported to be associated with mesenchymal-to-epithelial transition (MET), the reversed process of EMT [46,47,48]. In human metastatic breast model, VCAN derived from bone marrow myeloid progenitor cells induces MET of tumor cells in the premetastatic niche via inhibition of the TGFβ/SMAD2/3 pathway [46] (Table 1).

### 3.2. SLRPs: Diverse Regulatory Roles in Cancer Cell Signaling Related to Stemness and EMT

BGN has been associated with poor prognosis in several cancer types and seems to contribute to the EMT process [49,50,51,52,53,54]. In bladder cancer, BGN overexpression is associated with the EMT markers such as Snail, Slug, ZEB1-2, TWIST1-2, vimentin, and fibronectin, and negatively correlates with E-cadherin. BGN correlates with overall survival and the expression of the EMT markers N-cadherin and vimentin in head and neck squamous cell carcinoma patients [50]. BGN is further highly expressed in gastric cancer and has been associated with poor prognosis. Here, BGN expression is directly correlated with a mesenchymal gene signature. By knocking out BGN in gastric cancer cells, decreased levels of mesenchymal markers N-cadherin, ZEB1, ZEB2, Snail, NANOG, DPP4, and 3/4 October (POU5F1), and increased expression of the epithelial marker E-cadherin are observed. Treatment of KO cells with exogenous BGN leads to recovery of NANOG, but no differences in E-cadherin and N-cadherin expression levels are observed. Furthermore, the loss of BGN suppresses the colony formation capability of the cells [51]. BGN is a factor that participates in the integrated pathway of TGFβ/Snail with TNFα/NF-κB, which may facilitate tumor–stroma cross-talk during EMT in colorectal cancer [52] (Table 1). Furthermore, BGN enhances cancer cell stemness and EMT by affecting the interaction between cancer cells and mesenchymal stem cells during liver metastasis of colon cancer cells [53]. The expression of BGN in colon cancer cells is induced by high sugar concentration, fatty acids, and insulin, and its contact co-culture with mesenchymal stem cells. BGN overexpression in cancer cells is associated with phosphorylation of AKT, expression of mesenchymal markers, and liver metastasis [53].

Additionally, BGN appears to be highly expressed in cancer stem cells, such as colon and MCF-7-derived breast CSCs [33,54]. In the latter, depletion of BGN leads to decreased CSCs populations as shown by the reduction in the number of CD29 high, CD61+, and ALDH+ CSCs. BGN expression is required for the activation of NF-κB signaling pathway in breast CSCs as well as for increased tumorsphere forming ability, invasion, and metastasis (Table 1). Thus, BGN seems to contribute to the maintenance of breast CSC properties and is associated with worse prognosis in breast cancer [54]. On the contrary to the above, BGN is reported to have a tumor-suppressive function in pancreatic ductal adenocarcinoma, likely via trapping TGFβ within the ECM, hence decreasing the activation of the TGFβ/SMAD-independent pathway [55].

DCN exhibits a tumor-suppressive role as a pan-receptor tyrosine kinases inhibitor [56,57]. In normal gastric tissue, elevated levels of DCN have been reported, while in silico data and co-immunoprecipitation assays reveal its interaction with TGFβ. The interplay of asporin (ASPN), DCN, and TGFβ has been shown in a gastric adenocarcinoma dataset. Both ASPN and DCN interact with TGFβ and differentially regulate the activation of this pathway. DCN interacts with TGFβ in normal gastric epithelium, inhibiting the TGFβ canonical pathway [58]. Additionally, DCN inhibits the EMT-like phenotype in glioma through the inhibition of the c-Met/AKT/mTOR pathway and by inducing autophagy [59] (Table 1). In inflammatory breast cancer (IBC), DCN is significantly downregulated, while its overexpression suppresses cancer cell migration, invasion, and cancer cell stemness in vitro as well as cancer cell growth and metastasis in vivo. DCN physically interacts with E-cadherin and mediates its autophagic degradation and elimination, which in turn inhibits the E-cadherin/EGFR/ERK signaling axis [60]. Several treatment approaches based on DCN-mediated suppression of TGFβ and EMT have been proposed [61,62,63] (Table 1). For instance, the delivery of an oncolytic adenovirus expressing DCN blocks breast cancer cell growth and development of lung metastasis in a breast cancer mice model through multiple mechanisms involve inhibition of EMT, regulation of Wnt/β-catenin, VEGF and Met signaling as well as modulation of inflammatory and immune responses [61]. Furthermore, an oncolytic adenovirus co-expressing DCN and a soluble Wnt decoy receptor markedly prevents EMT and cancer cell metastasis in an orthotopic pancreatic xenograft tumor model [62]. DCN deficiency significantly promotes EMT in a DCN^−/−^ mice model of colitis-associated cancer. The intestinal epithelium of the DCN^−/−^ mice exhibits a marked increase in the protein levels of EMT markers including N-cadherin, vimentin, Snail, Slug, Twist, and MMP-2 and reduced levels of E-cadherin under unchallenged basal conditions. Induction of colitis-associated cancer further increases the expression of EMT-associated markers in DCN^−/−^ mice. Moreover, treatment with a combination of Celecoxib and DCN markedly inhibits EMT and proliferation of colorectal cancer cells in DCN^−/−^ mice and it is proposed as a potential adjuvant protein therapy [63].

ASPN is highly expressed by CAFs and plays important roles in cancer progression [64,65,66]. In pancreatic cancer, ASPN induces EMT in a paracrine manner via expression by pancreatic stellate cells. ASPN binds to CD44 in pancreatic cancer cells and activates the CD44/AKT/ERK-NF-kB signaling axis, which lead to EMT induction and increased migration and invasion [65] (Table 1). As mentioned above, in normal gastric tissue, DCN interacts with TGFβ, leading to tumor suppression. On the other hand, ASPN is increased in gastric cancer and can also interact with TGFβ. ASPN is thought to activate the SMAD2 pathway to induce EMT, as higher levels of phosphorylated SMAD2 have been observed in gastric cancer tissue compared with adjacent normal tissue [58] (Table 1). ASPN exhibits significantly increased expression levels in CAFs compared with normal fibroblasts. Scirrhous gastric cancer cells stimulate CAFs to express ASPN, which in turn causes increased invasion of both cell types in vitro and in vivo. The underlying mechanism includes the ASPN-mediated activation of Rac1 in both CAFs and cancer cells through its interaction with CD44 [67]. ASPN is also overexpressed in colorectal cancer tissues compared with adjacent normal tissues and its expression is associated with lymph node metastasis and TNM stage. In vitro experiments show that ASPN enhances migration and invasion of colorectal cancer cells. In colorectal cancer tissues, elevated co-expression of ASPN and p-cortactin (Tyr 241), an essential component for invadopodia formation and cellular migration, are found. When ASPN is overexpressed in HT-29 and LoVo colorectal cancer cells, the EGFR/Src axis is activated, leading to increased phosphorylation (Tyr 241) of cortactin. These data support that ASPN could be a biomarker and a potential therapeutic target for colorectal cancer [68]. Of interest, ASPN can be located intracellularly, acting as an EMT promoter. Cytoplasmic ASPN can recruit the TGFβ-induced p-SMAD2/3 on the nuclear membrane, facilitating their transportation to the nucleus inducing the expression of EMT-related genes, such as MMP-2, MMP-9, AHR, GLI2, ZEB1, TCF4, and TNC in colorectal cancer cells [69] (Table 1). Nevertheless, ASPN is expressed in the Hs578T breast cancer cell line, a cell line associated with a mesenchymal phenotype, and its expression can be regulated by BMP4, serum starvation, and 3D cultivation. Invasion of breast cancer cells is dependent on ASPN and it seems to be facilitated by CAFs [64]. Further studies verify that ASPN expression is low in non-tumor tissues, including breast tissue, on the contrary to DCN and BGN, which present high expression levels in normal tissues. In tumor tissues, ASPN is mainly detectable in the stroma secreted by CAFs and not by cancer cells, while the majority of breast cancer cells independently of their receptor status do not express ASPN. Normal breast fibroblasts can be stimulated to secrete ASPN by noninvasive luminal-like hormone receptor-positive cell lines such as T47D and MCF7 and not by highly metastatic triple-negative breast cancer cells such as MDA-MB-231 and MDA-MB-468 cells. This interesting observation is due to the secretion of IL-1β from triple-negative breast cancer cells, which blocks ASPN expression. Contrary to the previous studies, ASPN has also been found to be able to sequester TGFβ1, and thereby inhibiting the activation of SMAD2 and the subsequent promotion of EMT [70].

LUM is involved in multiple cancer-related processes, including EMT, and the impact of LUM in cancer aggressiveness is cancer-type specific [71,72]. In breast cancer cells, the expression of estrogen receptors ERα and ERβ is associated with EMT. Treatment of breast cancer cells with different expression of estrogen receptors with LUM induces the epithelial phenotype, especially in the highly invasive mesenchymal cells. These data indicate that LUM is a negative regulator of EMT and could be potentially used for breast cancer therapy [71,73,74]. In melanoma, the TF Snail triggers EMT, increases migration, invasion, and MMP-14 activity, while the inhibition of Snail-mediated EMT suppresses metastasis. LUM, through the suppression of elevated activity of MMP-14, decreases Snail-induced cell proliferation, migration, and invasion, and suppresses EMT (Table 1). Therefore, LUM could be applied for melanoma treatment [71,72,75,76]. LUM is transcriptionally suppressed by HMGA2, which is an oncogene which induces tumorigenesis and regulates specific genes and microRNAs associated with EMT, in ovarian cancer [77]. Glioblastoma and neuroblastoma cells increase the mRNA and protein levels of DCN and LUM when cultured under CSC enrichment conditions. The DCN+/LUM+ CSC-like glioblastoma and neuroblastoma cells form secondary neurospheres when cultured in anchorage-independent conditions, and present a quiescent, less proliferative phenotype and temozolomide resistance. It is possible that LUM and DCN play a dual role in the maintenance of CSCs and cell fate which is dependent on the tumor microenvironment (TME) [78].

### 3.3. SPOCK1 Is a Potent Inducer of EMT

The Testican/SPARC/Osteonectin, CWCV and Kazal-like domain (SPOCK) family of extracellular PGs is comprised of SPOCK1, SPOCK2, and SPOCK3. SPOCK1 has been shown to contribute to cancer cell invasion and metastasis through the promotion of EMT in multiple cancer types. Specifically, SPOCK1 is a downstream regulator of TGFβ and regulates Snail and Slug via the PI3K/AKT and Wnt/β-catenin signaling pathways, which lead to the regulation of the epithelial markers E-cadherin and ZO-1, and the mesenchymal markers vimentin and N-cadherin [79,80,81] (Table 1). SPOCK1 is upregulated in gastric [82], colorectal [83], lung [81], pancreatic [84], breast [85,86], esophageal squamous cell [87], and prostate cancer [88], as well as in glioblastoma [89], and it is associated with poor prognosis and EMT. In colorectal cancer, knockdown of SPOCK1 through shRNA results in increased levels of E-cadherin and decreased N-cadherin through the suppression of the PI3K/AKT pathway [83] (Table 1). Ectopic expression of SPOCK1 in epithelial cancer cell lines, including lung [81], pancreatic [84], breast [85,86], and esophageal squamous cell cancer [87], leads to the induction of EMT. In pancreatic cancer, SPOCK1 directly interacts with IκBα, thereby regulating NF-κB and ZEB2 [84] (Table 1). In breast cancer, the AKT/mTOR pathway is activated by SPOCK1 regulation of SIX1, and also induces cell proliferation and cell cycle progression [85]. In glioblastoma cells, SPOCK1 expression is positively correlated with the mRNA and protein levels of vimentin and N-cadherin as shown by gain or loss expression of SPOCK1, while SPOCK1 can induce temozolomide resistance [89]. HER2-positive gastric cancer cells acquire resistance to lapatinib via activation of c-Met and HER3 signaling. These cells exhibit an EMT phenotype with upregulated levels of SPOCK1. Inhibition of SPOCK1 restores sensitivity to lapatinib by suppressing c-Met and HER3 activation and the expression of β-catenin and promotes MET (Table 1). WNT/β-catenin signaling inhibition using XAV939, a small-molecule inhibitor, restores lapatinib sensitivity abrogated by SPOCK1 overexpression [82]. SPOCK1, due to its important roles in cancer, could serve as a potential therapeutic target. For instance, the flavonoid apigenin exerts an anti-metastatic effect in metastatic prostate cancer cell lines. Here, apigenin targets SPOCK1, which causes a reduction of Snail and Slug expression, further causing changes in vimentin, N-cadherin, and E-cadherin expression [88].

### 3.4. Versatile Functions of Pericellular PGs

AGRN has been shown to contribute to EMT in liver and pancreatic cancer [90,91,92]. In hepatocellular carcinoma (HCC) cells, AGRN is overexpressed and secreted in high levels compared with non-tumor liver cells. Furthermore, AGRN depletion leads to increased E-cadherin and reduced N-cadherin, vimentin, and Snail levels, while E-cadherin and vimentin levels are reversed after treatment with soluble AGRN, indicating that secreted AGRN can control the expression of EMT markers. AGRN through the LRP4/Musk axis activates FAK signaling and the Arp2/3 complex, which are critical for ECM degradation, cancer cell invasion, induction of EMT, and tumorigenesis [90] (Table 1). AGRN has also been associated with poor prognosis in patients with pancreatic ductal adenocarcinoma. Communication between cancer stem cells and non-stem cancer cells is mainly through extracellular vesicles that flow from CSCs to non-stem cancer cells. Extracellular vesicles intratumor communication network, which is formed through this procedure, contributes to the adaptation of cancer cells to microenvironmental changes and can lead to therapy resistance. Additionally, extracellular vesicles derived from CSCs have a distinct protein cargo, enriched in AGRN. AGRN-positive CSCs EVs enhance the activation of YAP through LRP4, promoting tumor growth (Table 1). Downregulation of AGRN in CSCs and in CSCs extracellular vesicles reduces tumor growth, and the use of a neutralizing anti-human AGRN antibody also reduces cell growth, as well as the activation of YAP. These results suggest that AGRN could be a therapeutic target in pancreatic cancer and, simultaneously, AGRN-positive CSCs extracellular vesicles can serve as prognostic biomarkers associated with increased risk for disease progression [92].

Collagen XVIII is a HSPG, associated with EMT of endothelial cells during endocardial cushions formation [93]. Endostatin is a fragment created by proteolytic cleavage of the carboxy-terminal non-collagenous domain of collagen XVIII and exerts anti-cancer properties [7]. In esophageal squamous cell carcinoma cells, recombinant human endostatin treatment combined with irradiation leads to the upregulation of PTEN and E-cadherin and inhibition of AKT/GSK-3β/Snail signaling axis and EMT, suggesting that this combination could be a potential approach for metastasis suppression [94] (Table 1). After treatment of basal carcinoma cells with endostatin, protein levels of the epithelial marker E-cadherin are increased, while N-cadherin, fibronectin, and vimentin are decreased, indicating that endostatin could induce MET [95]. In lung cancer, endostatin is used as an anti-angiogenic drug, but resistance to it has been observed. Endostatin therapy seems to increase the CSC population through the induction of TGFβ1 secretion and hypoxia, leading to endostatin resistance [96]. Endostar, a recombinant human endostatin, also has anti-tumor activity and suppresses EMT in ovarian and lung cancer [97,98]. Treatment of ovarian cancer cells with Endostar results in increased protein levels of E-cadherin and decreased N-cadherin, vimentin, Snail, MMP-2, and MMP-3. It is indicated that EMT suppression succeeds through blocking the expression of PD-L1 and the activation of STAT3, which are associated with tumor metastasis [97] (Table 1). Additionally, when A549 and H1975 lung cancer cells are cultured with supernatant of lung cancer-related polarized macrophages, EMT is induced. After co-treatment with Endostar and polarized macrophages, these alterations of EMT-related genes are restored, and it is proposed that Endostar-related inhibition of EMT occurs through the HGF-c-Met pathway [98]. Furthermore, in vitro experiments have shown that treatment of HCC cells with HYD-PEP06, an endostatin-derived synthetic polypeptide, inhibits metastasis by suppressing EMT through blocking of PI3K/AKT signaling pathway (Table 1). Simultaneously, HYD-PEP06 diminishes HCC CSCs metastasis via inhibition of the canonical Wnt/β-catenin pathway. These data suggest that HYD-PEP06 could be potentially used as a short peptide-agent for HCC treatment [99].

### 3.5. Syndecans: Dual Roles in EMT and Stemness

Syndecan-1 (SDC1) is a molecular biomarker for EMT during carcinogenesis. SDC1 mediates migration and adhesion through the interaction with GFs, and has a regulatory role on CSCs. Abnormal expression of SDC1 acts as a disease indicator in triple-negative breast [100,101], pancreatic [102], gastric, endometrial, and ovarian cancer [103]. SDC1 has emerged as a regulator of CSCs phenotype in triple-negative breast cancer [101], where SDC1 gene silencing in MDA-MB-231 cells [100] causes a downregulation in several components of the IL-6/STAT3, Notch, EGFR, and LRP6 pathways [100,101] (Table 1). Moreover, SDC1 holds a vital role in HCC sphere formation. The suppression of SDC1 induces the downregulation of the CSC markers CD133, CD44, and CD90, while the hepatoma spheres size is drastically decreased [104]. Numerous studies on different cancer types have characterized SDC1 as a key factor in EMT, as its repression in most carcinomas favors EMT [105]. In ductal breast cancers, the loss of SDC1 induces EMT, which can be utilized as a prognostic tool of survival [106]. Activation of the FGF-2/PI3K/AKT signaling axis promotes EMT in renal proximal tubular cell line HK2 via the overexpression of MMP-9 and heparanase (HPSE). These enzymes degrade SDC1 leading to sustained FGF-2 signaling [107] (Table 1). The FGF-2/SDC1/HPSE axis is also important for the regulation of EMT in pancreatic cancer [108]. This multifunctional role of SDC1 as a regulator of EMT was further investigated in prostate cancer, where the concurrent upregulation of ZEB1, a repressor of SDC1 expression [109], and Snail, and the downregulation of SDC1 induce a mesenchymal phenotype [110]. SDC1 knockdown also leads to increased proliferation, invasion, and migration of gallbladder cancer cells by regulating ERK/Snail signaling and induction of EMT [111] (Table 1). The suppression of SDC1 also induces EMT in oral cancer cells by activating the ERK/Snail signaling axis [112]. An association between EMT markers’ expression, such as SDC1, E-cadherin, and β-catenin, with reduced risk of poor outcome in colorectal cancer has been identified [113]. Finally, the addition of sphingosine-1-phospate in HCC cells induces EMT via an SDC1/MMP-7/TGFβ/HPSE mechanism. In more detail, S1P binds to its receptor and activates the PI3K/AKT and ERK1/2 signaling pathways. The PI3K/AKT pathway triggers HPSE, which further activates ERK1/2 and MMP-7. MMP-7 sheds SDC1, which leads to TGFβ1 production and EMT [114]. A correlation between EMT and nuclear translocated SDC1 has been observed in epithelial origin A549 adenocarcinoma cells. In more detail, during TGFβ-induced EMT in A549 adenocarcinoma cells, a loss of nuclear SDC1 is observed that is associated with EMT. Moreover, silencing of SDC1 leads to increased N-cadherin, nuclear localization of ZEB1, and invasion. The observed role of SDC1 strengthens the view about its involvement in modulation of EMP [115]. Finally, in invasive ductal carcinoma in situ cells, SDC1 gene silencing leads to the upregulation of several markers of EMT, such as the epithelial markers claudin and E-cadherin, the mesenchymal marker fibronectin, as well as the invasive markers MMP-3, MMP-9, and HPSE. These results may suggest a protective role of SDC1 in early stages of ductal carcinoma in situ, as its silencing leads to partial EMT accompanied by deregulation of ECM degradation enzymes [116].

Overexpression of syndecan-2 (SDC2) in HT29 colorectal cancer cells activates PKCγ and the FAK/ERK signaling pathway that induces the expression of MMP-7 involved in the shedding of E-cadherin [117,118] (Table 1). The overexpression of SDC2 in colorectal cancer results in induction of EMT and the upregulation of N-cadherin, Slug, Twist, vimentin, and MAPK signaling pathway activation, while SDC2 knockdown reduces cancer cell proliferation, migration, and invasion, and induces cell apoptosis [119]. Finally, circNFATC3, a circular RNA that is overexpressed in cervical cancer, works as a molecular sponge for miR-9-5p, which is a suppressor of SDC2 expression. As a result, the overexpression of circNFATC3 in cervical cancer leads to the overexpression of SDC2 and its downstream targets syntenin-1, NF-κB, and MMP-9 [120].

The data on the contribution of syndecan 3 (SDC3) in the induction of a mesenchymal phenotype are limited. Nevertheless, the receptor protein tyrosine phosphatase β/ζ seems to induce EMT in prostate cancer via cooperation with SDC3. In the early stages of prostate cancer, both molecules are expressed and the growth factor pleiotrophin binds to receptor protein tyrosine phosphatase β/ζ. As the cancer progresses, receptor protein tyrosine phosphatase β/ζ is downregulated and pleiotrophin binds to its second receptor, SDC3. This event triggers the induction of a mesenchymal phenotype via MAPK signaling pathway activation [121] (Table 1). SDC3 is associated with an EMT-stemness phenotype in ovarian cancer [122]. In particular, silencing of SDC3 reduces colony formation and 3D spheroid growth and stemness in ovarian cancer cells. Bioinformatic tools revealed numerous direct and indirect functional interactions of SDC3 with several stemness- and EMT-associated markers, such as E-cadherin, Snail, SDC1, and its involvement in multiple pathways such as Notch, Wnt, and Hedgehog [122].

Syndecan 4 (SDC4) has a universal expression in almost every cell type under physiological situations as well as in development and pathological conditions. SDC4 is localized at the focal adhesion sites interacting with a plethora of actin modulating molecules, such as PKCα, Rho GTPases, and FAK [123,124]. SDC4 is associated with aggressiveness in testicular germ tumors. The aggressive non-seminomatous testicular germ tumor cells present decreased levels of SDC4, which are further correlated with disease stage, lymph node metastasis, and vascular and lymphatic invasion. [125]. SDC4 levels are increased at the invasion front of colorectal cancer cells. SDC4 expression is significantly correlated with the pathological characteristics that indicate disease progression and inversely associated with disease-free and overall survival, meaning SDC4 emerges as a molecular marker of colorectal cancer cell aggressiveness [126]. Moreover, SDC4 is upregulated in TGFβ1-induced EMT in A549 lung adenocarcinoma cells. SDC4 knockdown results in recovered E-cadherin expression and decreased expression of mesenchymal markers vimentin and Snail; however, Slug protein expression is not affected by the SDC4 knockdown. To achieve a full epithelial morphology, both SDC4 and Slug need to be silenced, suggesting that Snail is a transcription factor downstream of SDC4, and that SDC4 regulates TGFβ1-induced EMT by cooperating with Slug [127]. In papillary thyroid cancer cells, the knockdown of SDC4 ameliorates their aggressive mesenchymal phenotype via the downregulation of Wnt/β-catenin signaling (Table 1). Additionally, the expression of SCD4 is positively associated with clinicopathological characteristics of papillary thyroid cancer patients, including tumor and lymph node metastasis, differentiation, and disease stage [128]. The mechanism behind the relation of SDC4 and Wnt/β-catenin is partially understood in Xenopus, where the molecule R-spondin 3, a modulator of Wnt, interacts with SDC4 and induces Wnt signaling via receptor complex endocytosis, resulting in the morphological differentiation of the cells [129]. To continue, chondroitin polymerizing factor was demonstrated to promote breast cancer aggressiveness via the structural alteration of SDC4 and especially addition of CS chains [130]. SDC4 further acts as a receptor for tenascin-C, an ECM molecule secreted from various bladder cancer cells. This interaction activates NF-κΒ and induces EMT, as shown by the expression of mesenchymal markers, such as Snail, N-cadherin, and vimentin [131] (Table 1).

### 3.6. Loss of Betaglycan (TGFBR3) Evokes EMT in Cancer Cells

The first extensive study in cancer about the role of TGFBR3 in EMT revealed that the deprivation of TGFBR3 expression can lead to increased invasion and migration of the pancreatic cancer cells PANC-1 without affecting Snail or E-cadherin levels. This is not mediated by its cytoplasmic domain or its co-receptor function, but is partially mediated by ectodomain shedding of TGFBR3, generating soluble TGFBR3 (sTGFBR3). TGFBR3 is downregulated during EMT and precedes downregulation of E-cadherin and cytoskeletal reorganization during the early stages of EMT. Surprisingly, the maintenance of TGFBR3 expression does not inhibit EMT, but instead suppresses the increased motility and invasiveness associated with the mesenchymal phenotype [38]. Both stimulations with TGFβ1 or BMPs repress expression of TGFBR3 in PANC-1 cells. In particular, BMPs cause the suppression of protein and mRNA levels of TGFBR3, which in basal conditions inhibits the phosphorylation of SMAD1. In EMT conditions, the BMP type I receptor ALK3 unhindered regulates p-SMAD1, which in turns activates the expression of MMP-2. The active form of MMP-2 seems to control the increased invasiveness observed in PANC-1 cells after the treatment with BMPs [132]. Moreover, TGFβ1 can also repress the expression of TGFBR3 in HCC cell lines, resulting in subsequent activation of the SMAD2 and AKT pathways and enhanced migratory and invasive cellular capacity [133].

Additionally, in oral squamous cancer, the simultaneous loss of TGFBR3 and growth differentiation factor 10/BMP3b induces EMT and chemotherapy resistance via the SMAD2 and ERK1/2 signaling pathways (Table 1). TGFBR3 is an upstream activator of growth differentiation factor 10 expression and they negatively regulate EMT, migration, and invasion via the same signaling pathways [134]. TGFBR3 can cooperate with a number of important miRNAs, including miR-19a, miR-424 [135], and miR-323b-3p [136]. In particular, in tongue squamous cancer miR-19a and miR-424 inhibit TGFBR3. TGFBR3 overexpression alone exerts a strong inhibitory effect on migration and EMT of CAL-27 tongue squamous cancer cells, while overexpression of miR-19a and miR-424 downregulates TGFBR3 and triggers EMT and cancer cell migration. Mechanistically, TGFBR3 directly interacts with β-arrestin 2, which in turns interacts with ΙκΒα, resulting in the inhibition of NF-κΒ signaling and migratory ability of CAL-27 cells [135] (Table 1). The implication of TGFBR3 in the inhibition of the NF-κB pathway and cellular migration via the interaction with β-arrestin 2 has also been demonstrated in MCF10A breast epithelial and MDA-MB-231 breast cancer cells [137]. MiR-323b-3p expression associates with the expression of TGFBR3 in an inverse manner. MiR-323b-3p is upregulated and TGFBR3 is downregulated in osteosarcoma to evoke tumorigenesis, with the opposite expression pattern being true in pulmonary metastasis where TGFBR3 acts as a tumor promoter for the development of pulmonary metastasis [136]. In murine mammary epithelial cells, TGFBR3 presents a basolateral localization which is directed by specific cytosolic residues to sustain an epithelial phenotype. Mutations of the proline 826 residue of TGFBR3 lead to apical localization of TGFBR3 that occurs before, and perhaps leads to the subsequent loss of epithelial cell polarity and promotes EMT independently of TGFβ ligand and ΤGFβR1 kinase activity. Apical localization of TGFBR3 can further lead to enhanced proliferation, migration, and invasion in vitro and enhanced tumorigenesis in an in vivo mouse model of breast carcinoma. The proper localization of TGFBR3 to the basolateral membrane domain is essential for the maintenance of epithelial cell polarity and suppression of breast cancer progression [138].

### 3.7. Glypicans: Contradictory Roles in EMT and cell Stemness

Data regarding the role of glypican 1 (GPC1) in the induction of EMT have revealed that GPC1 mainly interacts with TGFβ, FGF, and Wnt signaling pathways, all crucial regulators of EMT [37]. The pivotal role of GPC1 in EMT induction has been confirmed in several pathological conditions, such as esophageal carcinoma and colon adenocarcinoma. In particular, the overexpression of GPC1 in HT29 and HCT-116 colon cancer cells downregulates several epithelial markers, such as E-cadherin and upregulates vimentin, Snail, and Slug, leading to EMT. Moreover, GPC1 is negatively regulated via miR-96-5p and miR-149 [139]. Overexpression of Snail in MC38 colon adenocarcinoma cells induces EMT, and the Snail-overexpressing cells produce extracellular vesicles containing elevated quantities of GPC1 [140]. Interestingly, increased expression levels of GPC1 induce a mesenchymal phenotype in esophageal squamous cell carcinoma through the PTEN/AKT/β-catenin signaling pathway (Table 1). In particular, the overexpression of GPC1 reduces the levels and the activation of PTEN and increases the activation of AKT and the levels of β-catenin. GPC1 overexpression induces EMT as it eliminates the levels of E-cadherin and upregulates the expression of N-cadherin [141].

Glypican-3 (GPC3) displays diverse biological roles regarding tumor progression in a cell- and tissue-specific manner [7]. Several studies have outlined the role of GPC3 in the regulation of EMT, in a diverse number of cancer types, including breast [142] and HCC [143]. GPC3 is highly expressed in normal breast tissue and is markedly decreased in tumors. In breast cancer, GPC3 silencing in low metastatic MCF7 breast cancer cells stimulates an aggressive phenotype with EMT features; while, on the other hand, induction of its expression attenuates the aggressiveness of MDA-MB-231 cells, leading to MET. Part of the regulation of EMT characteristics from GPC3 is mediated by the inhibition of the Wnt/β-catenin signaling pathway and downregulation of ZEB1 [142] (Table 1). In another study, GPC3 re-expression in aggressive breast cancer cells returned mesenchymal-like breast cancer cells to an epithelial phenotype as shown by the suppression of Snail, ZEB1, vimentin, and increased expression of E-cadherin. It also impaired in vivo metastasis and induced tumor in vivo dormancy at the secondary tumor site through p38 MAPK signaling activation [144].

On the other hand, GPC3 evokes EMT in hepatocellular carcinoma, thereby displaying a tissue-specific dual role [7]. GPC3 knockdown in HCC HepG2 cells decreases proliferation, invasion, migration, and the expression of EMT-related molecules including MMP-2, MMP-9, Snail, Slug, and β-catenin, while it promotes the expression of E-cadherin as well as apoptosis [143]. GPC3-induced ERK activation promotes EMT and enhances cell migration and invasion in hepatocellular carcinoma [145] (Table 1). GPC3 and the transmembrane protein FAT1 directly interact and co-operate to evoke cell migration and EMT in HCC cell lines. Double silencing of those molecules regulates the expression of important EMT markers such as Snail, vimentin, and E-cadherin [146].

Several studies have shown the crucial role of glypican-4 (GPC4) as a mediator of stem cell maintenance, differentiation, self-renewal, and pluripotency. GPC4 is essential in ESCs and neural stem cells for the maintenance of progenitors and the response of ESCs to Wnt ligands [147,148]. In particular, the loss of GPC4 function leads to the acceleration of differentiation of mouse ESCs, as early markers of ectoderm, mesoderm, and endoderm differentiation are increased and SOX2 expression is decreased. Additionally, stem cells with GPC4 mutations are able to integrate into all three germ layers [147,148]. GPC4 is vital for the cell fate determination, pluripotency, tumorigenic potential, and Wnt pathway induction by downregulating GSK3 protein levels, as well as a number of other signal transduction pathways [147]. In neural SCs, GPC4 plays a key role in the astrocyte differentiation [149]. An important association is observed between GPC4 and 5-FU resistance in pancreatic cancer. GPC4 silencing results in the decrease of several stem markers, such as SOX2 and OCT4, and better response to 5-FU treatment, through the suppression of the Wnt/β-catenin signaling pathway (Table 1). GPC4 functions by facilitating the localization of Wnts on the cell membrane, thus activating Wnt/β-catenin signaling, which causes the increased chemoresistance and stem cell-like properties [150].

The suppressive role of glypican-5 (GPC5) in EMT has been described in lung adenocarcinoma [151] and prostate cancer [152]. Here, overexpression of GPC5 leads to the downregulation of EMT markers, such as vimentin and Snail, upregulation of E-cadherin, and inhibition of the Wnt/β-catenin signaling pathway, via the direct binding of GPC5 to Wnt3a (Table 1).

### 3.8. Serglycin Triggers Oncogenic Signaling and EMT

Serlgycin (SRGN) is the only intracellularly classified PG that can also be secreted by a number of different cell types, exerting crucial roles in the ECM and TME [40]. The role of SRGN in EMT and cancer cell stemness has been confirmed in a plethora of cancer types, such as HCC [153], breast cancer [154,155,156], colorectal [157], lung [158], and nasopharyngeal cancer [159]. SRGN also has an effect on tumor cell phenotype and stemness in glioblastoma [160]. SRGN is positively correlated with vimentin and negatively with E-cadherin, in HCC and nasopharyngeal carcinoma samples as well as in the nasopharyngeal carcinoma cell line CNE-2 and its highly metastatic clones [153,159]. Concerning breast cancer, knockdown of SRGN in mesenchymal-like MDA-MB-231 and the induction of the SRGN expression in epithelial MCF-7 cancer cells confirmed the vital role of SRGN in the induction of EMT [154,155]. High expression levels of SRGN facilitate EMT by upregulation of several ECM-degrading enzymes, activation of IL-8/CXCR2, and modulation of downstream signaling cascades including PI3K, SRC, and Rac [154] as well as the CD44/CREB1/TGFβ2 signaling axis, [155] (Table 1). SRGN is markedly upregulated during TGFβ-induced EMT [161]. SRGN/TGFβ2 interlay establishes a positive feedback loop that promotes EMT in breast cancer cells. TGFβ2 binds to TGFβR, activating SMAD2/3 that promotes the expression and secretion of SRGN. Then, secreted SRGN binds to CD44 on the breast cancer cell membrane, triggering intracellular signals activating CREB1 that increase the expression of TGFβ2 [155]. Importantly, epithelial breast cells are more responsive to TGFβ-induced EMT when expressing SRGN, as serglycin CRISPR/Cas9 knockout delays the onset of EMT upon TGFβ treatment. Nevertheless, after the induction of EMT, the suppression of SRGN by siRNA knockdown could not revert the acquired mesenchymal phenotype. Moreover, during the EMT process, the expression of SRGN is regulated by ZEB1 in mammary epithelial cells [161]. Knockout of SRGN has no impact on the growth of primary tumor or the number of mammary tumors formed in the MMTV-PyMT-driven mouse breast cancer model. In contrast, the ablation of SRGN completely inhibits lung metastasis. E-cadherin expression is significantly higher in the SRGN-deficient primary tumors, indicating a more epithelial cancer cells phenotype [162]. SRGN further plays an important role in chemoresistance and stemness of breast cancer cells. Specifically, SRGN via the activation of the ITGA5/FAK/CREB/YAP signaling axis establishes a positive feedback loop that upregulates SRGN and HDAC2 and prompts cell stemness [156] (Table 1). The SRGN/CD44 interaction was thoroughly investigated in A549 and H460 lung cancer cell lines [158]. SRGN interacts with CD44, which serves as a stem-cell marker, via its CS residues, activating NF-κΒ and upregulating claudin-1 expression, which disturbs tight cell junctions, leading to EMT and increased cell stemness [158] (Table 1). Furthermore, in colorectal cancer, SRGN transcriptional overexpression is associated with a mesenchymal phenotype, as N-cadherin and vimentin are upregulated and E-cadherin is downregulated through induction by the hypoxia-inducible factor 1a [157]. SRGN is also a mediator of cell stemness and glioblastoma to astrocytic differentiation. Suppression of SRGN in LN-18 glioblastoma cells triggers the downregulation of several pluripotency and self-renewal markers, such as SOX2, OCT4, LIF, MSI1, NES, KLF4, Nanog, and ALDH-1, while it simultaneously upregulates the expression of astrocyte differentiation factors including GFAP, SPARCL-1, and Snail. Moreover, LN-18 glioblastoma cells with suppressed levels of SRGN exhibit a low ability for colony formation and a reduction of tumorigenesis in vivo [160].

## 4. Conclusions

PGs are important structural and functional constituents of ECMs that participate in matrix organization and cell–cell and cell–matrix interactions. Their expression is often deregulated in tumor cells and the surrounding stroma, providing a provisional matrix for tumor cell growth and spread. Recent studies show PGs as potent players in the landscape of cancer cell signaling and plasticity, as PGs are implicated in the regulation of EMT and cancer cell stemness by intervening in a variety of signaling pathways. PGs exhibit complicated roles as they often can control cancer cell stemness and EMT in a cell- and context-dependent manner. The elucidation of the underlying molecular mechanisms may provide us with new therapeutic tools for cancer treatment.

## Figures and Tables

**Table 1:** Main roles of PGs in cancer cell EMT and stemness.

PG	Localization/Family	Function	Mechanism
**VCAN**	Extracellular/Hyalectans	Induction of EMT (V1 isoform)	Activation: EGFR/AKT/GSK3β axis
		Inhibition of EMT (V2 isoform)	Inhibition: EGFR/AKT/GSK3β axis TGFβ/SMAD2/3 pathway
		Induction of stemness	Activation: EGFR/AKT/GSK3β axis
**BGN**	Extracellular/SLRPs	Induction of EMT	Cooperate with TGFβ/Snail—TNFα/NF-κB pathways
		Induction of stemness	Activation: NF-κB signaling
**DEC**	Extracellular/SLRPs	Inhibition of EMT	Inhibition: Receptor tyrosine kinases c-Met/AKT/mTOR/autophagy TGFβ signaling
**ASPN**	Extracellular/SLRPs	Induction of EMT	Activation: CD44/AKT/ERK-NF-kB axis TGFβ/SMAD2/3 signaling
**LUM**	Extracellular/SLRPs	Inhibition of EMT	Inhibition: MMP-14/Snail axis
**SPOCK1**	Extracellular/SPOCK	Induction of EMT	Activation: PI3K/AKT signaling Wnt/β-catenin signaling NF-κB/ZEB2 axis c-Met/HER3/β-catenin
**AGRN**	Pericellular	Induction of EMT	Activation: LRP4/Musk/FAK/Arp2/3 axis LRP4/YAP axis
**COL XVIII/ENDOSTATIN**	Pericellular	Inhibition of EMT	Inhibition: AKT/GSK-3β/Snail axis PI3K/AKT signaling PD-L1/STAT3 axis
**SDC1**	Cell membrane/Syndecans	Induction of stemness	Regulation: IL-6/STAT3, Notch, EGFR, LRP6 pathways
		Inhibition of EMT	Inhibition: FGF-2/PI3K/AKT signaling ERK/Snail axis
**SDC2**	Cell membrane/Syndecans	Induction of EMT	Activation: PKCγ/FAK/ MAPK signaling
**SDC3**	Cell membrane/Syndecans	Induction of EMT	Activation: Pleiotrophin/MAPK signaling
**SDC4**	Cell membrane/Syndecans	Induction of EMT	Activation: Wnt/β-catenin axis Tenascin-C/NF-κΒ axis
**BETAGLYCAN/ TGFBR3**	Cell membrane	Inhibition of EMT	Inhibition: SMAD2/AKT/ERK1/2 signaling NF-κΒ signaling
**GPC1**	Cell membrane/Glypicans	Induction of EMT	Activation: AKT/β-catenin axis
**GPC3**	Cell membrane/Glypicans	Inhibition of EMT (Breast cancer)	Inhibition: Wnt/β-catenin/ZEB1 axis
		Induction of EMT (HCC)	Activation: ERK signaling
**GPC4**	Cell membrane/Glypicans	Induction of stemness	Activation: Wnt/β-catenin axis
**GPC5**	Cell membrane/Glypicans	Inhibition of EMT	Inhibition: Wnt/β-catenin axis
**SRGN**	Intracellular (Matrix secreted molecule)	Induction of EMT/stemness	Activation: IL-8/CXCR2 signaling CD44/CREB1/TGFβ2 signaling ITGA5/FAK/CREB/YAP axis CD44/NF-κΒ/claudin axis

## Abbreviations

AGRN	Agrin
ASPN	asporin
BGN	biglycan
BMP	bone morphogenetic protein
CAFs	cancer-associated fibroblasts
CSCs	cancer stem cells
CS	chondroitin sulfate
CTGF	connective tissue growth factor
DCN	decorin
DS	dermatan sulfate
EBs	embryoid bodies
ESCs	embryonic stem cells
EGFR	epidermal growth factor receptor
EMP	epithelial mesenchymal plasticity
EMT	epithelial-to-mesenchymal transition
ECMs	extracellular matrices
FGF	fibroblast growth factor
FOXC2	forkhead box C2
GAGs	glycosaminoglycans
GPC1	glypican 1
GPC3	glypican-3
GPC4	glypican-4
GPC5	glypican-5
GFs	growth factors
HCC	hepatocellular carcinoma
HPSE	heparanase
HS	heparan sulfate
HP	heparin
HA	hyaluronan
IBC	inflammatory breast cancer
IGFR-I	insulin-like growth factor receptor I
ILs	interleukins
KS	keratan sulfate
LUM	lumican
MMPs	matrix metalloproteinases
MET	mesenchymal-to-epithelial transition
PRRX1	paired-related homeobox 1
PDGF	platelet-derived growth factor
PGs	proteoglycans
SRGN	serglycin
SLRPs	small leucine-rich proteoglycans
SDC1	syndecan-1
SDC2	syndecan-2
SDC3	syndecan 3
SDC4	syndecan 4
SPOCK	Testican/SPARC/Osteonectin CWCV and Kazal-like domain
TGFBR3	TGFβ receptor III
TLRs	toll like receptors
TFs	transcription factors
TGFβ	transforming growth factor β
TAMs	tumor-associated macrophages
TME	tumor microenvironment
VEGFA	vascular endothelial growth factor A
VCAN	versican
ZEB	zinc finger E-box binding homeobox
